# Increase in Short-Interval Intracortical Facilitation of the Motor Cortex after Low-Frequency Repetitive Magnetic Stimulation of the Unaffected Hemisphere in the Subacute Phase after Stroke

**DOI:** 10.1155/2015/407320

**Published:** 2015-04-28

**Authors:** Eduardo Arruda Mello, Leonardo G. Cohen, Sarah Monteiro dos Anjos, Juliana Conti, Karina Nocelo F. Andrade, Fernanda Tovar Moll, Theo Marins, Corina A. Fernandes, Waldyr Rodrigues, Adriana Bastos Conforto

**Affiliations:** ^1^Neurology Clinical Division, Clinics Hospital/São Paulo University, Avenida Dr. Enéas C. Aguiar 255/5084, 05403-010 São Paulo, SP, Brazil; ^2^Human Cortical Physiology and Stroke Rehabilitation Section, National Institutes of Neurological Disorders and Stroke, National Institutes of Health, Building 10, Room 7D54, Bethesda, MD 20892, USA; ^3^Institute of Biomedical Sciences and National Center for Structural Biology and Bioimaging (CENABIO), Federal University of Rio de Janeiro, Avenida Carlos Chagas Filho 373, Prédio do CCS, Bloco M, Cidade Universitária, 21941-902 Rio de Janeiro, RJ, Brazil; ^4^D'Or Institute for Research and Education (IDOR), Rua Diniz Cordeiro 30, 22281-032 Rio de Janeiro, RJ, Brazil; ^5^Instituto de Ensino e Pesquisa, Hospital Israelita Albert Einstein, Avenida Albert Einstein 627/701, 05601-901 São Paulo, SP, Brazil

## Abstract

Low-frequency repetitive transcranial magnetic stimulation of the unaffected hemisphere (UH-LF-rTMS) in patients with stroke can decrease interhemispheric inhibition from the unaffected to the affected hemisphere and improve hand dexterity and strength of the paretic hand. The objective of this proof-of-principle study was to explore, for the first time, effects of UH-LF-rTMS as add-on therapy to motor rehabilitation on short-term intracortical inhibition (SICI) and intracortical facilitation (ICF) of the motor cortex of the unaffected hemisphere (M1_UH_) in patients with ischemic stroke. Eighteen patients were randomized to receive, immediately before rehabilitation treatment, either active or sham UH-LF-rTMS, during two weeks. Resting motor threshold (rMT), SICI, and ICF were measured in M1_UH_ before the first session and after the last session of treatment. There was a significant increase in ICF in the active group compared to the sham group after treatment, and there was no significant differences in changes in rMT or SICI. ICF is a measure of intracortical synaptic excitability, with a relative contribution of spinal mechanisms. ICF is typically upregulated by glutamatergic agonists and downregulated by gabaergic antagonists. The observed increase in ICF in the active group, in this hypothesis-generating study, may be related to M1_UH_ reorganization induced by UH-LF-rTMS.

## 1. Introduction

Maladaptive plasticity in the form of excessive inhibition of the affected hemisphere by the unaffected hemisphere can negatively impact recovery of the paretic hand after stroke. A single session of low-frequency repetitive transcranial magnetic stimulation of the unaffected hemisphere (UH-LF-rTMS) can downregulate excitability of the motor cortex of the unaffected hemisphere (M1_UH_), reflected by decrease in amplitude of motor evoked potentials (MEPs) recorded from the nonparetic hand in patients in the chronic phase after stroke [1]. Decrease in MEP amplitude may be related to long-term-depression-like (LTD-like) plasticity [2]. In addition, UH-LF-rTMS decreases the duration and amplitude of the ipsilateral silent period, a measure of interhemispheric inhibition recorded by delivering transcranial magnetic stimulation (TMS) to M1_UH_ during voluntary muscle contraction of the paretic hand [1].

Importantly, improvements in motor function of the paretic hand were reported after single [1, 3, 4] or several [5–11] sessions of UH-LF-rTMS, applied either alone or in combination with motor training, in the subacute or chronic stages after stroke. These encouraging results suggest that UH-LF-rTMS has potential as an adjuvant therapeutic strategy in stroke rehabilitation.

Despite these exciting findings, mechanisms underlying effects of UH-LF-rTMS are not entirely clear. There is virtually no information about effects of UH-LF-rTMS on M1_UH_ short-interval intracortical inhibition (SICI) or facilitation (ICF), in the subacute stage after stroke. SICI and ICF probably reflect intracortical excitability in separate excitatory and inhibitory neurons, with a relative contribution of spinal mechanisms to ICF. SICI is likely mediated by GABAa receptors activity, while ICF is mainly influenced by the glutamatergic system (NMDA receptors) [12–14]. The objective of this pilot, double blinded, randomized proof-of-principle study was to explore, for the first time, effects of UH-LF-rTMS as an add-on therapy to standard-of-care rehabilitation, on SICI and ICF of M1_UH_, in patients within 5–45 days after ischemic stroke and mild to severe hand paresis.

## 2. Methods

The protocol was conducted as part of a study that compared effects of UH-LF-rTMS or sham rTMS as add-on therapies to standard-of-care rehabilitation [11].

### 2.1. Participants

**Figure 1:**
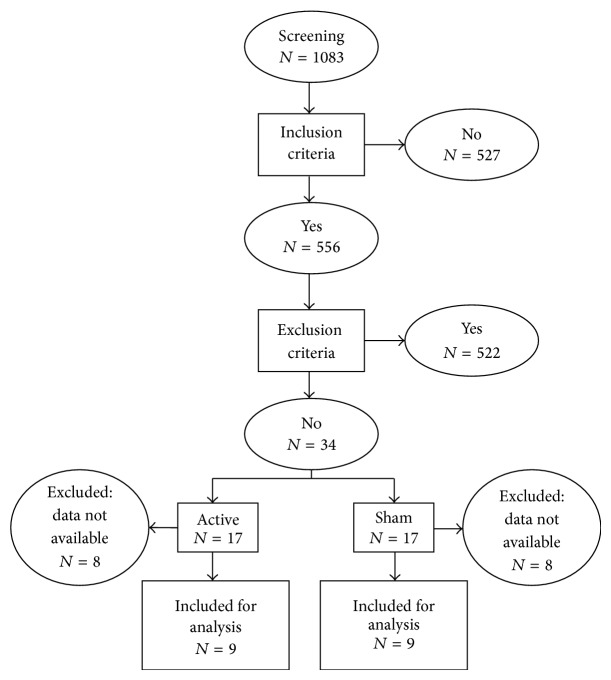
Flow chart.

Between February 2008 and October 2012, 1083 patients with ischemic stroke were screened for the study. The flow chart is shown in Figure 1. Inclusion criteria were age, 18–80 years; first-ever symptomatic ischemic stroke involving up to 50% of the internal carotid artery territory, confirmed by CT or MRI; time from stroke, 5–45 days; and mild to severe hand paresis (Medical Research Council scale, 4-0 in finger flexion or extension).

Exclusion criteria were previous symptomatic strokes; history of seizures; other neurologic diseases and uncontrolled chronic diseases, such as congestive heart failure or cancer; shoulder pain; joint deformity of the paretic upper limb; and relative or absolute contraindications to TMS [15]: use of implantable infusion pumps, pacemaker, pregnancy, intracranial hypertension, intracranial metal, bone skull defects, and use of drugs that can interfere on cortical excitability such as tricyclic antidepressants, neuroleptics, or benzodiazepines. In addition, an implanted stent in the carotid or vertebral arteries less than two months was considered a relative contraindication to TMS. Other exclusion criteria were inability to provide informed consent due to severe aphasia, anosognosia, or cognitive impairment as well as inability to attend to treatment sessions. The protocol was approved by the local Ethics Committee, and all patients provided informed consent to participate. The protocol was registered in Clinicaltrials.gov (NCT01333579).

One patient in the sham group had a recurrent stroke on the day before the second session of treatment and did not complete the study. Due to technical problems (malfunction of the paired-pulse TMS stimulator), cortical excitability data in 7 patients in the active group and 7 patients in the sham group were not collected. One patient in the active group coughed repeatedly during one of the TMS experiments. Because contraction of facial muscles can interfere on cortical excitability [16], data from this patient were excluded. Therefore, data from 18 patients (9 per group) were analyzed.

### 2.2. Baseline Measures

The following characteristics were evaluated at baseline: age, gender, handedness before stroke (Oldfield inventory) [17], days after stroke, infarct side/location, NIH stroke scale [18], and modified Rankin scale. Infarcts were classified as corticosubcortical or subcortical according to involvement of primary motor, primary somatosensory, supplementary motor, or premotor cortices on MRI (16) or CT (2) by a radiologist blinded to group assignment.

### 2.3. Experimental Design

Patients were randomized in blocks by the principal investigator with a basic random number computerized generator in a 1 : 1 ratio to receive immediately before rehabilitation treatment lasting for 60 minutes, either active or sham rTMS, five days per week, during two weeks (total, 10 treatment sessions). Patients were unaware of group assignment.

### 2.4. rTMS Intervention

In each treatment session, the optimal site of motor stimulation of the UH (“hot spot”) was defined as the location where TMS elicited the largest MEPs in the nonparetic* abductor pollicis brevis* muscle (APB) with surface electrodes. The signal was amplified and filtered (10 Hz to 2 kHz) with an electromyography (EMG) and evoked potential measuring unit (MEB-9104 J, Nihon Kohden, Japan).

In both groups, 1 Hz rTMS was administered with a figure-of-eight coil (MCF B-65) at 90% of the nonparetic APB muscle resting motor threshold (rMT) with a biphasic MagPro compact stimulator (Alpine Biomed), for 25 minutes (1500 pulses). For the active intervention, TMS coil was tangentially positioned by an investigator on the nonparetic APB “hot spot” in the UH with the intersection of both wings at 45° angle with the midline. In the sham intervention, the coil was held perpendicularly to the vertex. All patients were comfortably seated, with their arms in a constant relaxed position, wore earplugs, and were instructed to remain at rest during TMS.

### 2.5. Outcome Measures

TMS was delivered to the UH at the nonparetic APB “hot spot” through a figure-of-eight shaped coil (mean diameter 70 mm), connected to a Bi-Stim 200^2^ module (MagStim, UK). The coil was held by an investigator. EMG activity was recorded from surface electrodes placed over the unaffected APB. EMG responses were amplified (×1000), filtered (2 Hz–2 kHz), sampled at 5 kHz and recorded on a computerized data acquisition system built with the LabVIEW graphical programming language [19]. Its conditional triggering feature was used to deliver TMS stimuli only when the APB muscle was relaxed. Relaxation of APB muscle was defined as EMG activity at baseline <50 *μ*V peak-to-peak amplitude for at least 1 second [19]. Trials showing EMG activity ≥50 *μ*V peak-to-peak amplitude were excluded during recordings. Vigilance was continuously monitored by an investigator sitting in front of the patient. This investigator was also in charge of running the LabVIEW acquisition system and saving data files throughout the experiment.

The following TMS measurements were performed before the first (D0) session of treatment and after the last (D10) session of treatment in patients in both groups:

- rMT, defined as the minimum TMS intensity required to elicit at least three out of six MEPs ≥ 50 microV in consecutive trials [20, 21];
- SICI and ICF measured with paired-pulse TMS [22, 23]. The conditioning stimulus (CS) intensity was set to 80% of the unaffected APB rMT. The intensity of the test stimulus (TS) was that required to evoke MEPs of approximately 0.5 to 1 mV. The order of presentation of inhibitory (2 ms), excitatory (10 ms), and control (test stimulus alone) trials was randomized. Eighteen trials were recorded for each interstimulus interval. Results are expressed as average percentages of MEP amplitudes in conditioning trials and in test trials.

Raw data from paired-pulse experiments were inspected by an investigator blinded to group assignment with a playback application in system built with the LabiVIEW graphical programming language (sampling rate 5000 Khz) [19].

### 2.6. Statistical Analysis

This was an exploratory study and determination of the sample size was not formally determined to evaluate TMS outcomes. Data are presented as means (±standard deviations) or as medians and intervals. Distribution was checked with the Shapiro-Wilk test. Normally distributed data were analyzed with parametric tests. Nonnormally distributed data were analyzed with nonparametric tests. Baseline characteristics were compared with Mann-Whitney or Fisher's exact tests. Between-group differences in changes in rMT, SICI, and ICF (baseline − after treatment, D0 − D10) were compared with Mann-Whitney tests. *P* values ≤0.05 were considered statistically significant.

## 3. Results

There were no significant differences in baseline characteristics between the active and sham groups (Table 1). There were no serious adverse events. In the active group, two patients reported headache or local pain (22.2%), two patients reported nuchal pain (22.2%), and five patients reported drowsiness (55.6%). In the sham group, three patients reported headache or local pain (33.3%), four patients reported nuchal pain (44.4%), and six patients reported drowsiness (66.7%).

**Figure 2:**
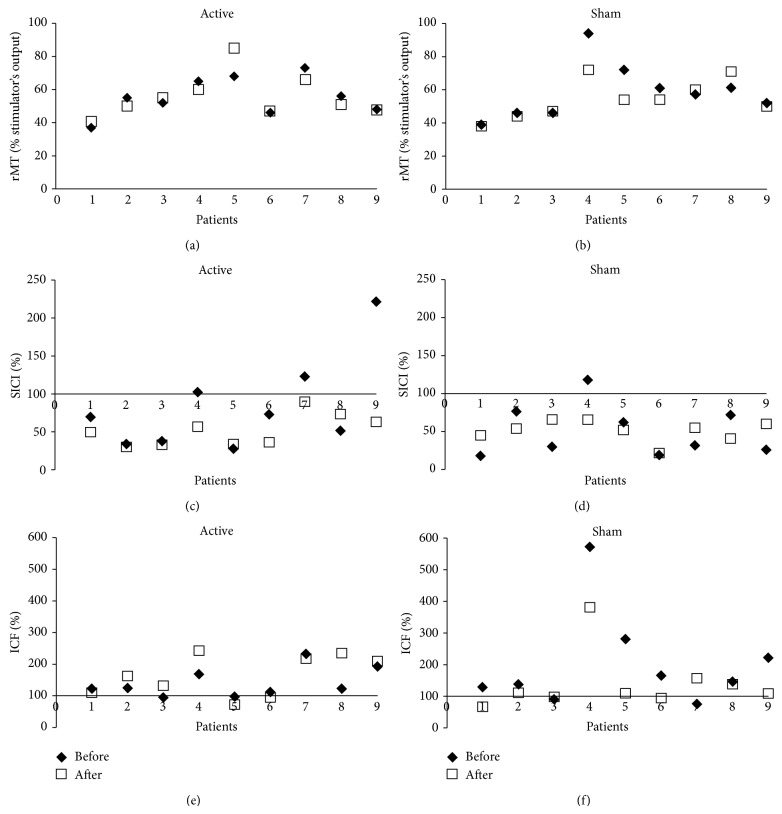
Differences in changes in rMT, SICI, and ICF between the sham and active groups. rMT = resting motor threshold; SICI = short-interval intracortical inhibition; ICF = short-interval intracortical facilitation.

Results are shown in Figure 2 and Table 2. There was a significant increase in ICF in the active group compared to the sham group after treatment (*P* = 0.038). Five patients in the active group and only one patient in sham group presented increase in ICF after treatment. ICF decreased in five subjects in the sham group.

There were no significant differences in changes in rMT (*P* = 0.489) or SICI (*P* = 0.145) after treatment between the two groups. Post hoc analysis showed that the power to find significant differences between the two groups was 20% for rMT and 34% for SICI.

One of the subjects (Patient 4 of the sham group in Figure 2) presented a higher level of ICF (571.8%) compared to other subjects, at baseline. We did not find an objective reason to exclude this subject from the analysis. However, post hoc analysis was performed without this subject, and the difference in change in ICF between the two groups was no longer statistically significant (*P* = 0.074).

## 4. Discussion

The main result of this study was the increase in ICF in M1_UH_ after 10 sessions of UH-LF-rTMS in patients with hand paresis, in the subacute phase after ischemic stroke. ICF increased in the active group and decreased in the sham group. Both groups received customary rehabilitation during the protocol.

This is the first time that a change in ICF in the UH is reported after UH-LF-rTMS. ICF is a measure of intracortical synaptic excitability, with a relative contribution of spinal mechanisms. ICF is usually upregulated by glutamatergic agonists [21]. Because ICF has a spinal component, a spinal mechanism underlying the observed change cannot be excluded.

The observed increase in ICF in M1_UH_ in the active group is consistent with results obtained in patients at a chronic stage (average, 31 months after stroke), after 10 sessions of constraint-induced therapy (CIT) [24]. CIT consists of restraint of the unaffected upper limb during waking hours and intensive and repetitive task-oriented therapy of the affected upper extremity, to overcome reliance on the nonparetic upper limb to perform daily activities, and consequent “learned nonuse” of the paretic limb.

It is possible that changes in the M1_UH_ synaptic excitability may reflect reorganization induced by specific interventions such as rTMS or CIT. Reorganization in M1_UH_ does not necessarily translate into measurable, relevant motor improvement of the paretic upper limb. For instance, modified CIT (shaping training of the paretic hand, with or without restraint of the upper limb for two weeks) is associated with increased cortical thickness in M1_UH_ [25]. However, increased thickness does not correlate with enhancement of motor function evaluated with the Wolf Motor Function Test or the Motor Activity log. The link between structural or functional changes in M1_UH_ and behavioral improvements remains to be clarified.

A single session of LF-rTMS was reported to* decrease* ICF in the stimulated hemisphere in healthy subjects, when a similar paired-pulse protocol was applied by Romero et al. [26]. The opposite result observed in patients with stroke may reflect a difference in experimental paradigm (one rTMS session in the study by Romero et al., in contrast with ten sessions in the present study) or a state-dependent phenomenon; that is, effects of rTMS may vary according to baseline excitability of the stimulated cortex [27].

We did not include a control group to investigate differences in baseline excitability between patients with stroke and controls. The two studies that compared ICF in the UH of patients in the subacute phase after stroke, and ICF in hemispheres of healthy subjects, did not report significant differences [4, 28]. Still, comparable ICF at baseline should not necessarily translate into comparable responsiveness to ten sessions of rTMS.

The lack of significant changes in rMT or SICI may reflect absence of changes in membrane excitability mediated by ion channels, as well as absence of modulation of gabaergic synapses mediating SICI, by 1 Hz rTMS of M1_UH_. Alternatively, it may reflect insufficient power given the study's sample size.

Still, the main limitation of this study is the number of patients. After screening 1083 patients, 34 patients fulfilled criteria to participate in the study. This low rate of inclusion is consistent with those reported in other studies of TMS in stroke [29]. Then, because of technical problems with the stimulator, data from 14 patients were not available. Post hoc analysis showed that if results from one patient were excluded, the results would no longer be statistically significant. Nevertheless, we did not find an objective reason to exclude this subject. Furthermore, the number of subjects included in the analysis was not substantially different from those reported by other studies that evaluated excitability to TMS in patients with stroke [4, 28]. Considering that SICI and ICF vary considerably even in healthy subjects, multicenter studies may be necessary to overcome challenges in this field of research.

Another limitation of this study is the lack of evaluation of interhemispheric inhibition after UH-LF-rTMS. In patients with mild hand paresis in the chronic phase after stroke, interhemispheric inhibition of the affected hemisphere by the UH, evaluated by the ipsilateral silent period, decreases after UH-LF-rTMS [1]. Recording of the ipsilateral silent period in this context involves stimulation of the UH during voluntary contraction of the paretic hand. We could not record the ipsilateral silent period because we included patients with severe hand motor deficits, unable to perform hand muscle contractions in the paretic hand, as required to record the ipsilateral silent period while applying TMS to the M1_UH_. Finally, spinal excitability was not evaluated.

In summary, the increase in ICF in M1_UH_ after ten sessions of active UH-LF-rTMS indicates that modulation of synaptic excitability in the UH or in the spinal cord is associated with this intervention. Further studies are required to confirm this result in a larger sample of patients with stroke and to investigate the functional role of this phenomenon, possibly related to reorganization, in stroke recovery.

## Figures and Tables

**Table 1:** Characteristics of the patients.

Characteristics	Active (*n* = 9)	Sham (*n* = 9)	*P* value
Age (mean ± standard deviation)	57.1 ± 11.8	51.1 ± 17.7	0.438^1^
Gender (men/women)	5/4	4/5	1.0^2^
Handedness (oldfield inventory) (%)	82.3 (42.9–100)	62.5 (0–100)	0.345^3^
Days after stroke	27 ± 3.6	30.1 ± 10	0.582^1^
Affected hemisphere (right/left)	5/4	3/6	0.637^2^
Infarct location (corticosubcortical/subcortical)	4/5	6/3	0.637^2^
NIH Stroke Scale (median, interval)	5.3 (4–8)	5.9 (1–11)	0.964^3^
Modified Rankin Scale	3 (2–5)	3 (0–4)	0.639^3^
Baseline rMT (%)	55.5 ± 3.8	58.6 ± 5.5	0.859^3^
Baseline SICI (%)	82.2 ± 20.3	50.5 ± 11.3	0.145^3^
Baseline ICF (%)	140.3 ± 15.5	202.3 ± 50.7	0.402^3^
Baseline PEM_TS_	739.1 ± 355.0	804.1 ± 639.5	0.965^3^

*N* = number; rMT = resting motor threshold; SICI = short-interval intracortical inhibition; ICF = short-interval intracortical facilitation; ^1^Unpaired *t*-test. ^2^Fisher's exact test. ^3^Mann-Whitney test.

**Table 2:** Measurements of excitability to transcranial magnetic stimulation.

Measure	Baseline	After treatment	Difference	*P* value
rMT				
Active	55.9 (37–73)	55.9 (41–85)	0.3 (−7–17)	0.478^1^
Sham	58.7 (39–94)	54.4 (38–72)	−4.2 (−22–10)	
SICI				
Active	82.3 (27.9–221.5)	51.9 (30.1–89.7)	−30.4 (−158.7–−52.3)	0.145^1^
Sham	50.5 (17.8–117.8)	51.0 (21.5–65.5)	0.4 (−52.3–35.5)	
ICF				
Active	140.3 (94.6–231.1)	164.1 (72.0–242.6)	23.8 (−23.8–112.4)	0.038^1∗^
Sham	202.3 (75.8–571.8)	140.7 (66.8–380.9)	−61.6 (−190.9–81.3)	

rMT = resting motor threshold; SICI = short-interval intracortical inhibition; ICF = short-interval intracortical facilitation; mean; interval. ^1^Mann-Whitney test, ^*^ *P* ≤ 0.05 (Mann-Whitney test).
